# Regulation of Cytokine Production by the Unfolded Protein Response; Implications for Infection and Autoimmunity

**DOI:** 10.3389/fimmu.2018.00422

**Published:** 2018-03-05

**Authors:** Judith A. Smith

**Affiliations:** ^1^Department of Pediatrics, University of Wisconsin-Madison School of Medicine and Public Health, Madison, WI, United States; ^2^Department of Medical Microbiology and Immunology, University of Wisconsin-Madison School of Medicine and Public Health, Madison, WI, United States

**Keywords:** unfolded protein response, endoplasmic reticulum stress, infection, virus, bacteria, autoimmunity, cytokine regulation, autoinflammatory disease

## Abstract

Protein folding in the endoplasmic reticulum (ER) is an essential cell function. To safeguard this process in the face of environmental threats and internal stressors, cells mount an evolutionarily conserved response known as the unfolded protein response (UPR). Invading pathogens induce cellular stress that impacts protein folding, thus the UPR is well situated to sense danger and contribute to immune responses. Cytokines (inflammatory cytokines and interferons) critically mediate host defense against pathogens, but when aberrantly produced, may also drive pathologic inflammation. The UPR influences cytokine production on multiple levels, from stimulation of pattern recognition receptors, to modulation of inflammatory signaling pathways, and the regulation of cytokine transcription factors. This review will focus on the mechanisms underlying cytokine regulation by the UPR, and the repercussions of this relationship for infection and autoimmune/autoinflammatory diseases. Interrogation of viral and bacterial infections has revealed increasing numbers of examples where pathogens induce or modulate the UPR and implicated UPR-modulated cytokines in host response. The flip side of this coin, the UPR/ER stress responses have been increasingly recognized in a variety of autoimmune and inflammatory diseases. Examples include monogenic disorders of ER function, diseases linked to misfolding protein (HLA-B27 and spondyloarthritis), diseases directly implicating UPR and autophagy genes (inflammatory bowel disease), and autoimmune diseases targeting highly secretory cells (e.g., diabetes). Given the burgeoning interest in pharmacologically targeting the UPR, greater discernment is needed regarding how the UPR regulates cytokine production during specific infections and autoimmune processes, and the relative place of this interaction in pathogenesis.

## Introduction: Immune Sensing of Danger and Endoplasmic Reticulum (ER) Stress

How does the immune system sense pathogenic threats and respond appropriately? Cells in the immune system “see” the environment in little snippets: adaptive immune cells such as T cells bear surface receptors triggered by major histocompatibility complexes (MHC) loaded with peptides 8–20 amino acids in length (1). Even within these short stretches, the T cell receptor may physically interact with only five amino acids (2, 3). Antibodies, constituting the B cell receptors, also recognize similarly small molecules, averaging 18–19 contact residues (up to 5 contiguous) (4). Innate immune cells, the first responders on the scene of infection, including neutrophils, macrophages, and dendritic cells, express pathogen-sensing receptors on their surfaces, inside their endosomes and cytosol collectively referred to as pattern recognition receptors (PRRs). These PRRs recognize conserved molecular arrays on pathogens, or pathogen-associated molecular patterns (PAMPs) such as bacterial lipopolysaccharide (LPS), flagellin and lipoproteins, β-glucans and mannans on yeast, and nucleic acids from viruses. The nucleic acid sensors detect types of nucleic acids that are not normally produced (e.g., dsRNA) or located in unusual settings, such as dsDNA in the cytosol or single stranded RNA in endosomes. Classes of PRRs include the toll-like receptors (TLRs), C-type lectin receptors, nucleotide-binding domain and leucine-rich repeat containing receptors (NLRs), retinoic acid inducible gene I (RIG-I) family helicases, and other cytosolic nucleic acid sensors (5–8).

When the outside world is observed in small pieces, the specificity of immune receptors becomes problematic. For instance, pathogens may express peptides with identical or functionally analogous amino acids stretches as endogenous peptides, a phenomenon referred to as “molecular mimicry” (2, 9). The classic example is the antibody cross-reactivity between streptococcal *N*-acetyl-beta-d-glucosamine and the cardiac myosin protein (10). This specificity issue, complicating the discrimination of self and infectious non-self, led to the “danger theory” put forth by Polly Matzinger in 1994, and then later refined over the years, that the immune system responds to challenges in accordance with contextual clues from damaged tissues (11–13). These damage-associated signals have been termed “danger-associated molecule patterns” (DAMPs). Different types of DAMPs have been reviewed recently in Ref. (14). When tissue is damaged, and cells destroyed by necrosis rather than apoptosis, specific molecules are released into the surrounding milieu. Examples of released products include dramatic increases in extracellular ATP, extracellular nucleic acids such as double-stranded DNA, mitochondrial DNA, chaperones such as high-mobility group box 1 (HMGB1) and heat shock proteins, interleukins IL-1α and IL-33, and uric acid (15, 16). Even in the absence of actual cellular destruction, infection or stress-triggered calcium signaling, and the generation of reactive oxygen species (ROS) may be considered DAMPs. During infections, the generation of multiple DAMPs provides the context to signal significant organismal insult.

The “danger” hypothesis was initially conceived to address issues with adaptive immune (T and B cell) self-non-self-discrimination. However, this same conceptual requirement for damage that provides context for dendritic cell activation and T cell stimulation may also help with several other specificity issues in innate immunity. Consider the microbiome: humans are widely covered on external and internal surfaces with trillions of microbes that constitute our natural microflora. Microbial-associated molecular patterns (MAMPs) also stimulate PRRs. For instance, theoretically, the same TLR4 that recognizes LPS on an invading pathogen could also be triggered by gut gram-negative bacteria. However, in the absence of tissue damage or stress, the healthy steady-state microbiome does not normally trigger inflammatory responses.

Many damage-generated endogenous products, such as extracellular matrix proteins, also stimulate PRRs (17). Indeed, the same PRRs poised to recognize PAMPs/MAMPs do “double duty” and respond to DAMPs, a testament to natural efficiency and repurposing (14). Alternatively, it has been suggested that pathogens have evolved to take advantage of PRRs evolutionarily aimed at wound repair (12). As an example of endogenous product recognition, the nucleic acids released by dying cells that are taken up into endosomes stimulate endosomal TLRs. The same non-specificity inherent in TLR4 that enables recognition of a broad variety of LPS structures may also allow TLR4 to respond to endogenous products such as fibrinogen or HMGB1 (18). The dual recognition of endogenous products and pathogens by the same receptors again poses a problem of specificity, as non-infectious damage (that releases DAMPs) may not merit an anti-pathogen response. How does the immune system determine whether to mount a wound healing response or an inflammatory response? Is there a titration by numbers or types of DAMPs (and PAMPs) and does this discrimination occasionally fail? Even in the absence of pathogens, “sterile” damage may liberate significant endogenous ligands for PRRs. One example of an over-exuberant inflammatory response in the face of sterile damage is the post-traumatic inflammatory response syndrome that occurs in the absence of inciting infection (16). Aberrant recognition of endogenous products may also drive non-resolving wound responses that lead to fibrosis (19).

Vance et al. have proposed that pathogenic organisms provide extra contextual clues that alert the immune system (20). The immune system recognizes certain bacterial products produced only by living (rather than dead), invasive pathogenic bacteria, so-called “vita PAMPs”: for instance, live bacteria produce cyclic-di-nucleotides second messengers that activate the host cytosolic stimulator of interferon gene (STING) (8, 21). Access to the cytosol may be the factor that provides the key information. As an example, the lysteriolysin O that enables *Listeria* release into the cytosol is required for immunogenicity (20). Other bacterial pathogens contain secretion systems that provide a conduit between vacuoles and host cytosol. Release of products *via* this route (e.g., flagellin) may then trigger cytosolic inflammasome sensors (22, 23). Disruption of the cytoskeleton may also be directly sensed by the host cell. The mechanisms by which this occurs remain unclear, but may involve co-localization of PRRs (NOD proteins and inflammasome components) with the actin cytoskeleton (24, 25).

**Figure 1:**
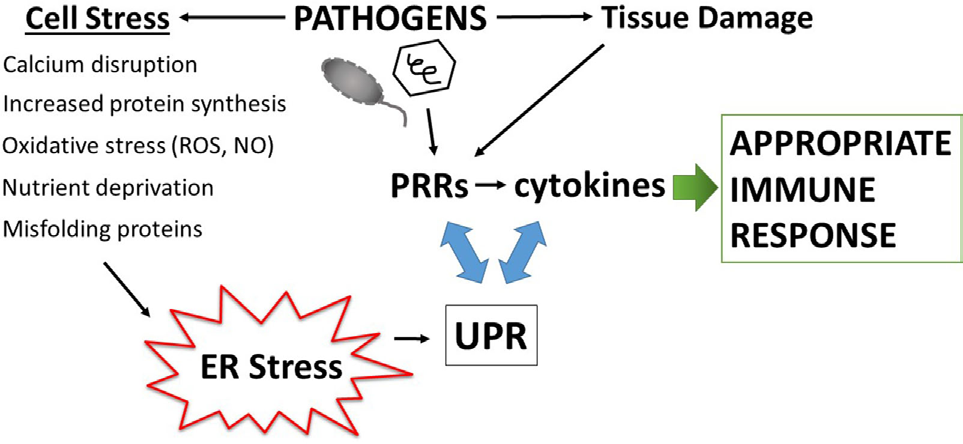
Amplification of pathogen immune responses *via* endoplasmic reticulum (ER) stress and the unfolded protein response (UPR). Pathogens cause tissue damage, intracellular host stress, and stimulate pattern recognition receptors (PRRs) which then induce cytokines. Multiple pathogen-triggered cellular insults cause stress in the ER that impacts protein folding and thus induces the UPR. The UPR, PRR activation, and cytokine production intersects on multiple levels (see main text and Figure 3), with interactions going in both directions (blue two-sided arrows). This amplification mechanism generates an immune response commensurate with the degree of pathogenic threat.

Disruption of fundamental cellular processes such as protein production, may also contribute to immune calibration, titrating up the threat level either appropriately, as in the case of infection, or inappropriately in autoimmunity. All cells must make protein to survive. Secreted and transmembrane proteins are manufactured in the ER. Amazingly, the ER accomplishes protein folding in a very crowded environment, estimated at 100 mg/ml, a concentration that could theoretically promote aggregation (26). The ER is also the site of sterol and phospholipid synthesis and the major cellular store for calcium. Indeed, many of the protein folding chaperones, including the carbohydrate-binding calnexin and calreticulin, immunoglobulin heavy chain binding protein (BiP/Grp78), and protein disulfide isomerases require high concentrations of calcium for their function (27). The formation of intermolecular and intramolecular disulfide bonds during protein folding generates ROS. Thus, to maintain redox equilibrium, the ER contains buffering anti-oxidant enzymes. Related to the exigencies of the folding process, a broad variety of environmental stressors may adversely impact protein folding, such as decreased glucose or amino acids, hypoxia, decreases in ER calcium, excessive reactive oxygen radicals, increased demands in protein production, as well as the presence of misfolding proteins. To safeguard protein production and ensure quality control, ER-stress triggers the activation of several biochemical pathways collectively referred to as the unfolded protein response (UPR). The UPR restores proteostasis equilibrium by increasing capacity (ER size and chaperone production) as well as decreasing protein client load, through translational inhibition and the process of ER-associated peptide degradation (ERAD). If ER stress becomes irremediable (excessively severe or prolonged), the UPR initiates apoptosis. One could envision how infections result in the multiple ER stresses listed above: viral infections dramatically increase protein production; bacteria consume nutrient resources and stimulate oxygen radical production. Because of the universal need for protein production, and the sensitivity to a wide variety of environmental or internal stressors, the UPR is well poised to sense pathogenic danger and transduce the stress signal into a heightened immune response (Figure 1).

The UPR plays a physiologic role by enabling the function of highly secretory cells such as hepatocytes, plasma cells, and acinar or islet pancreatic cells. For example, mice deficient in key UPR components die early of pancreatic insufficiency and diabetes (28, 29). The UPR also supports the development of specific cells in the immune system. Even before B cells ramp up antibody production to become plasma cells, the UPR is engaged by the plasma cell differentiation program (30–32). Similarly, optimal development and survival of dendritic cells, and differentiation of eosinophils requires the UPR (33, 34). Not only does the UPR support the development of specific immune cells, but it also globally shapes the immune responses in many cell types (32, 35).

Over the past 10–15 years, it has become apparent that one way in which the UPR tunes immune responses is through the modulation of cytokine production (35). Cells of the immune system communicate *via* cytokines, which are soluble secreted proteins encompassing the families of interleukins, interferons (IFNs), and tumor necrosis factor (TNF) family members among other mediators (36). Both the magnitude and types of cytokines produced program the immune response to respond appropriately to different types of threats. For instance, type I IFN-α/β induce hundreds of target genes aimed at containing and eliminating viral invasion. Cytokines such as TNF-α, IFN-γ, and IL-6 promote inflammatory innate responses that enhance anti-bacterial activities. IL-4, IL-5, and IL-13 heighten anti-parasitic immunity. On the other hand, IL-10 and transforming growth factor β (TGF-β) limit immune destructiveness and collateral damage to the host by toning down innate and adaptive immune responses. Following the sensing of danger signals *via* PRRs or adaptive immune leukocyte receptors, inflammatory pathways are set in motion that culminate in the activation of cytokine-regulatory transcription factors. Intriguingly, UPR pathways interweave through all levels of cytokine regulation: the UPR impacts the PRRs that sense pathogenic molecules, downstream inflammatory signaling pathways, and ultimately, the activation of cytokine-regulatory transcription factors, such as nuclear factor kappa-light-chain-enhancer of activated B cells (NF-κB), activator protein 1 (AP-1), and the interferon regulatory factors (IRFs). This interaction between the UPR and inflammation is a “two-way street”; in certain tissues (e.g., the liver), inflammatory cytokines induce ER stress, setting up the potential for a positive feedback loop (37). The modulation of cytokine production by intracellular stress during infection has implications for how the immune system detects and responds appropriately to pathogens. The drawback to anti-pathogen cytokine augmentation is the potential for inappropriate boosting of immune responses resulting in autoimmunity. Below, the variety of mechanisms linking the UPR with cytokine production and the implications of this interaction for infection and autoimmunity will be addressed.

## The UPR

The metazoan UPR comprises three primary signaling pathways stemming from the activation of ER-stress sensors inositol requiring enzyme 1 (IRE1), activating transcription factor 6 (ATF6), and protein kinase R (PKR) like endoplasmic reticulum kinase (PERK) (27, 38). These three sensors reside in the membrane of the ER, poised to respond to stressors that increase the abundance of unfolded proteins. In their inactive state, the stress sensors associate with the folding chaperone BiP. When ER luminal load of unfolded proteins increases, BiP releases the sensors to preferentially bind hydrophobic patches on misfolded protein, thus resulting in the activation of the three pathways. In addition to this BiP “titration” model, alternative mechanisms of activation have been described: crystallographic resolution of the yeast IRE1 revealed an ER luminal structure that forms an MHC-like peptide-binding groove upon dimerization, thus potentially allowing direct sensing of unfolded peptides (39). The mammalian pocket is too narrow to accommodate peptides, but may undergo a conformational change upon activation by peptide binding (40, 41). Although the PERK luminal domain has high-structural homology with IRE1, direct peptide binding has not been described for PERK. On the other hand, significant alterations in lipid content of the ER (e.g., increased acyl chain saturation) may also directly activate IRE1 and PERK, independently of their ER luminal domains (42).

Inositol requiring enzyme 1 is the most evolutionarily conserved ER stress sensor, and the only UPR pathway present in single cell organisms such as yeast. In mammals, the IRE1α (*ERN1*) isoform is ubiquitously expressed, whereas IRE1β (*ERN2*) is restricted to mucosal epithelial surfaces such as the lung and gut (43, 44). The cytosolic portion of IRE1 contains two functional domains: a kinase domain and an endonuclease domain. Upon sensing unfolded protein, IRE1 dimerizes and auto-trans phosphorylates, a prerequisite for activation. Intriguingly, the IRE1 endonuclease has only one specific mRNA target, known as Hac-1 in yeast and XBP1 in higher eukaryotes. IRE1 cleaves a 26 base pair loop out of the XBP1 mRNA, causing a frame shift mutation that removes a premature stop codon. The “unspliced” XBP1 mRNA encodes a shorter unstable protein with DNA binding domain only, but the longer “spliced” XBP1 mRNA encodes the full length transcription factor with DNA binding and transcriptional transactivating domains (45). XBP1 increases the production of folding chaperones (e.g., ERdj4), components involved in ERAD and increases phospholipid synthesis and ER size (31, 46, 47). By increasing ER capacity and decreasing ER client load, XBP1 is considered a largely “adaptive” pro-life response (48, 49). In addition to splicing XBP1, upon prolonged or severe stress, IRE1 may non-specifically degrade mRNAs in proximity to the ER in a process termed regulated IRE1-dependent decay (RIDD) of mRNA (50, 51). This non-specific endonuclease process is thought to decrease ER protein client load, as many of the degraded mRNAs encode proteins in the secretory pathway. XBP1 splicing and the RIDD functions of IRE1 may be experimentally dissociated, but the precise mechanisms governing the switch between these activities remain elusive (52). Degree of IRE1 oligomerization may regulate RNase substrate preference (53). The IRE1 kinase domain associates with other molecules in a multi-molecular complex referred to as the “UPRosome” (45, 54). Through association with TNF-receptor-associated factor 2 (TRAF2) and apoptosis signal-regulating kinase 1 (ASK1), IRE1 phosphorylates c-Jun N-terminal kinase (JNK), thus linking ER stress with autophagy, apoptosis, and inflammatory signaling (described more below) (55). Intriguingly, IRE1 also associates with the pro-apoptotic B cell lymphoma 2 (Bcl2)-family members Bcl2-antagonist/killer 1 (Bak) and Bcl2-associated X protein (Bax), which, through unknown mechanisms, enhance IRE1 kinase activity (56).

Protein kinase R like endoplasmic reticulum kinase oligomerizes and trans phosphorylates early during the UPR. PERK phosphorylates eukaryotic initiation factor 2α (eIF2α) on serine 51, thus inhibiting the guanine nucleotide-exchange activity of eIF2B required for recycling eIF2α to its GTP-bound form (57). By this mechanism, PERK inhibits ribosomal function and globally diminishes protein translation of capped mRNAs. This decrease in protein production is essential for stress adaptation, in that interference with eIF2α phosphorylation leads to proteotoxicity during ER stress (58). Certain mRNAs with inhibitory upstream short open reading frames such as the mRNA encoding the transcription factor ATF4 are preferentially translated when eIF2α is phosphorylated (59, 60). ATF4 stimulates the production of a pro-apoptotic transcription factor C/EBP homologous protein (CHOP). Together CHOP and ATF4 achieve most of the transcriptional program stemming from PERK activation, which includes the induction of proteins involved in amino acid transport, autophagy, folding chaperones, and redox regulatory proteins in addition to pro-apoptotic molecules (61, 62). ATF4 also initiates relief of the translational blockade through induction of growth arrest and DNA damage-inducible 34 (GADD34). GADD34 forms a complex with, and activates protein phosphatase 1, which dephosphorylates eIF2α (63). Thus, the global translational decrease is transient. Other molecules also impact eIF2α phosphorylation status: as an example of cross-talk between UPR pathways, XBP1 regulates p58^IPK^ which binds and inhibits PERK, thus promoting eIF2α dephosphorylation (46, 64, 65). During the non-stressed state, constitutive repressor of eIF2α phosphorylation maintains eIF2α dephosphorylation (66). Interestingly, other molecules with PERK homology, such as PKR, general control non-derepressible 2 (GCN2), and Heme-regulated eIF2α kinase also phosphorylate eIF2α, in response to dsRNA, amino acid deprivation, and heme deficiency, respectively, thus broadening the scope of stressors utilizing this response pathway. For this reason, the eIF2α pathway has also been referred to as the “Integrated Stress Response” pathway (61, 67). In addition to eIF2α, PERK also phosphorylates nuclear factor erythroid 2 (Nrf2), freeing it from the Kelch-like ECH associated protein 1 inhibitory protein. PERK thus enables Nrf2 nuclear translocation and an increase in anti-oxidant protein production (68).

**Figure 2:**
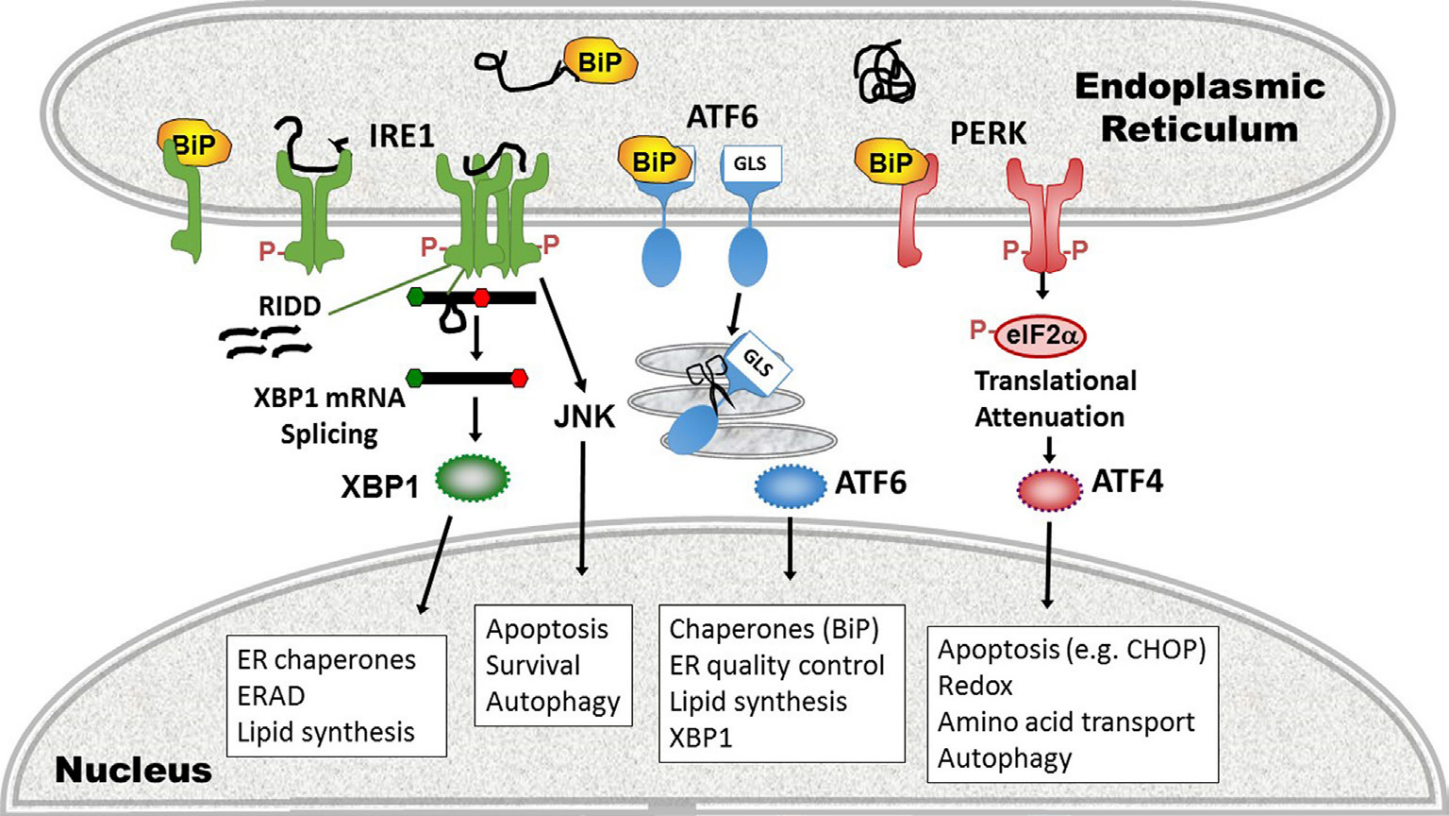
Three pathways of the unfolded protein response (UPR). (1) inositol requiring enzyme 1 (IRE1) pathway (left, green), a dual endonuclease and kinase, binds the chaperone binding protein (BiP) in its monomeric state. On sensing unfolded/misfolded protein IRE1 oligomerizes and auto-trans phosphorylates (red Ps). Activation of the endonuclease specifically splices 26 nucleotides out of the XBP1 mRNA, causing a frameshift mutation that removes a premature stop codon, thus enabling translation of the full length transcription factor. With increased stress, the non-specific endonuclease function cleaves endoplasmic reticulum (ER)-associated mRNAs in a process called regulated IRE1-dependent decay (RIDD). The IRE1 kinase domain associates with other signaling partners that phosphorylate Jun N-terminal kinase (JNK). ERAD, ER-associated degradation. (2) Activating transcription factor 6 (ATF6) pathway (middle, blue): ATF6 release of BiP uncovers a Golgi localization signal (GLS) enabling translocation to the Golgi. There it is cleaved by Site-1 and Site-2 proteases (scissors), liberating the ATF6 transcription factor. (3) Protein kinase R like endoplasmic reticulum kinase (PERK) pathway (right, pink): in the presence of phosphorylated protein, PERK also oligomerizes and transphosphorylates, activating its kinase activity. PERK in turn phosphorylates eIF2α, resulting in transient global translational inhibition apart from a few specific mRNAs such as ATF4. ATF4 promotes transcription of the apoptosis-inducing transcription factor C/EBP homologous protein (CHOP). Cellular processes altered by the UPR pathways and key gene targets that are UPR components are in boxes.

In the third major UPR pathway, release of BiP from ATF6 uncovers a Golgi localization signal, enabling translocation of ATF6 from ER to Golgi (69). In the Golgi, Site-1 and Site-2 proteases cleave ATF6, liberating the active transcription factor. ATF6 also has two isoforms, ATF6α, and ATF6β. Most of the UPR-related activity is dependent upon ATF6α, but there is some redundancy required for development as deletion of both in mice is embryonic lethal (70). ATF6 binds ER stress element sites by itself or as a heterodimer with XBP1; thus, there is some overlap in function. ATF6 also upregulates *XBP1* message, another instance of UPR pathway cross-talk (71). Certain UPR target gene chaperones, such as glucose-regulated protein 94 and BiP itself are primarily ATF6-dependent (70). Besides ATF6, in specific cell types, ER stress regulates Site-1 cleavage of other basic leucine zipper transcription factor proteins [e.g., Cyclic AMP-responsive element-binding protein H (CREBH), old astrocyte specifically induced substance, CREB4] (72). In the liver, CREBH participates in inflammatory responses by activating the production of C-reactive protein and serum amyloid P components of the acute phase response (37). For a basic summary of the three UPR pathways, see Figure 2.

## Mechanisms of Cytokine Regulation by ER Stress

Activation of the UPR is sufficient to induce low levels of inflammatory cytokine production, even in the absence of ostensible infectious stimuli or PRR ligation. In one of the earliest studies to note this phenomenon, a 2005 study by Li et al., free cholesterol loading of macrophages induced ER stress-dependent mitogen-activated protein (MAP) kinase signaling and NF-κB activation, resulting in production of IL-6 and TNF-α (73). Subsequently, studies using classic pharmacologic UPR inducers such as tunicamycin and thapsigargin have also noted low level “sterile” inflammatory cytokine production (74, 75). ER stress induces inflammatory cytokines by modulating inflammatory signaling cascades, activating “canonical” cytokine-regulatory transcription factors, as well as *via* the actions of the UPR-activated transcription factors themselves.

All three UPR pathways impact the activation of NF-κB. In the quiescent state, NF-κB family members (p50, p52, p65, RelB, and c-Rel) reside in the cytoplasm, bound to inhibitory factor κB (IκB). Upon immune signaling, IκB kinase (IKK) phosphorylates IκB, targeting it for ubiquitination and proteolytic destruction. The degradation of IκB permits NF-κB to translocate into the nucleus where it induces inflammatory cytokines such as IL-6 and TNF-α (76). IRE1 increases basal IKK activity *via* TRAF2, promoting NF-κB translocation (77–79). IRE1 may also promote NF-κB activation indirectly *via* regulation of glycogen synthase kinase 3 (80, 81). The IκBα protein has a shorter half-life compared with NF-κB, thus the PERK-dependent global translational shutdown preferentially affects IκB expression levels over NF-κB, leaving NF-κB free to translocate (82). Downstream of PERK, CHOP also enhances NF-κB signaling *via* transcriptional repression of the negative regulator peroxisome proliferator-activated receptor (83). ATF6 impacts NF-κB activation through a pathway involving mammalian target of rapamycin signaling and protein kinase B (Akt) dephosphorylation (84, 85). Finally, the calcium dysregulation and ROS generated during ER stress may contribute to NF-κB activation, either by enhancing induction of UPR pathways or other mechanisms (86). NF-κB regulates cytokine production in conjunction with other transcription factors such as the AP-1 heterodimer of Fos and Jun family transcription factors. MAP kinases [e.g., p38, extracellular regulated kinase (ERK) and JNK] regulate the activation of AP-1 factors (87). ER stress intersects with MAP kinase signaling in multiple ways [reviewed in Ref. (88)]: IRE1 promotes the activation of AP-1 family members *via* ASK-1 mediated JNK and p38 phosphorylation (55, 89). ERK phosphorylation during ER stress is also partially IRE1-dependent (90). In bronchial epithelial cells, PERK and ATF6 promote ERK and p38 signaling, and in cholesterol loaded macrophages, CHOP was required for ERK activation (73, 91). P38 positively feeds back on the UPR, phosphorylating CHOP and ATF6, and thus increasing their activities (92–94).

In addition to the classic pro-inflamamtory cytokines, the UPR regulates type I IFN. IFN-β is one of the earliest IFNs produced in response to viral infection and PRR engagement, and by binding the type I IFN receptor (IFNAR) and upregulating IRF7, promotes the production of multiple IFN-α species and induction of the full anti-viral interferon program (95). In the *ifnb1* promoter “enhanceosome” region, NF-κB, AP-1, and IRF3 bind cooperatively to initiate transcription (96–98). Like NF-κB, unactuated IRF3 remains cytoplasmic. Upon phosphorylation on multiple serines and threonines, IRF3 dimerizes and translocates into the nucleus where it binds its gene targets (99). IRF3 phosphorylation also enables association with the transcriptional co-activator CREB-binding protein (CBP/p300) (100). ER stress induces IRF3 phosphorylation and nuclear translocation, although the precise mechanisms are not yet clear and may depend upon the type of stress. Calcium disruption (as through the SERCA pump inhibitor thapsigargin) and oxygen glucose deprivation activate IRF3 through a STING-dependent mechanism, whereas agents that disrupt N-linked glycosylation (e.g., tunicamycin) appear to utilize a STING-independent, but Site-1/Site-2 protease (ATF6?)-dependent pathway (101).

In addition to the activation of canonical inflammatory transcription factors and IRFs, the classic UPR transcription factors which orchestrate the UPR bind directly to genetic cytokine-regulatory elements. Through chromatin precipitation analyses, XBP1 was detected at the promoters of the IL-6, and TNF-α encoding genes, a *tnf* enhancer, as well as an enhancer element downstream of the *ifnb1* gene (74, 102). In response to short chain fatty acids, ATF4 (downstream of PERK and the integrated stress response) binds the cAMP response element in the *Il6* promoter (103). CHOP binds the IL-23p19 (*Il23A*) promoter in dendritic cells in response to LPS, ER stress, and most fully to the combination of LPS and ER stress (104). On the other hand, certain UPR-regulated transcription factors such as ATF3 have anti-inflammatory effects, and may play a role in regulating pathogen responses, ischemic preconditioning, and cancer (105–109).

**Figure 3:**
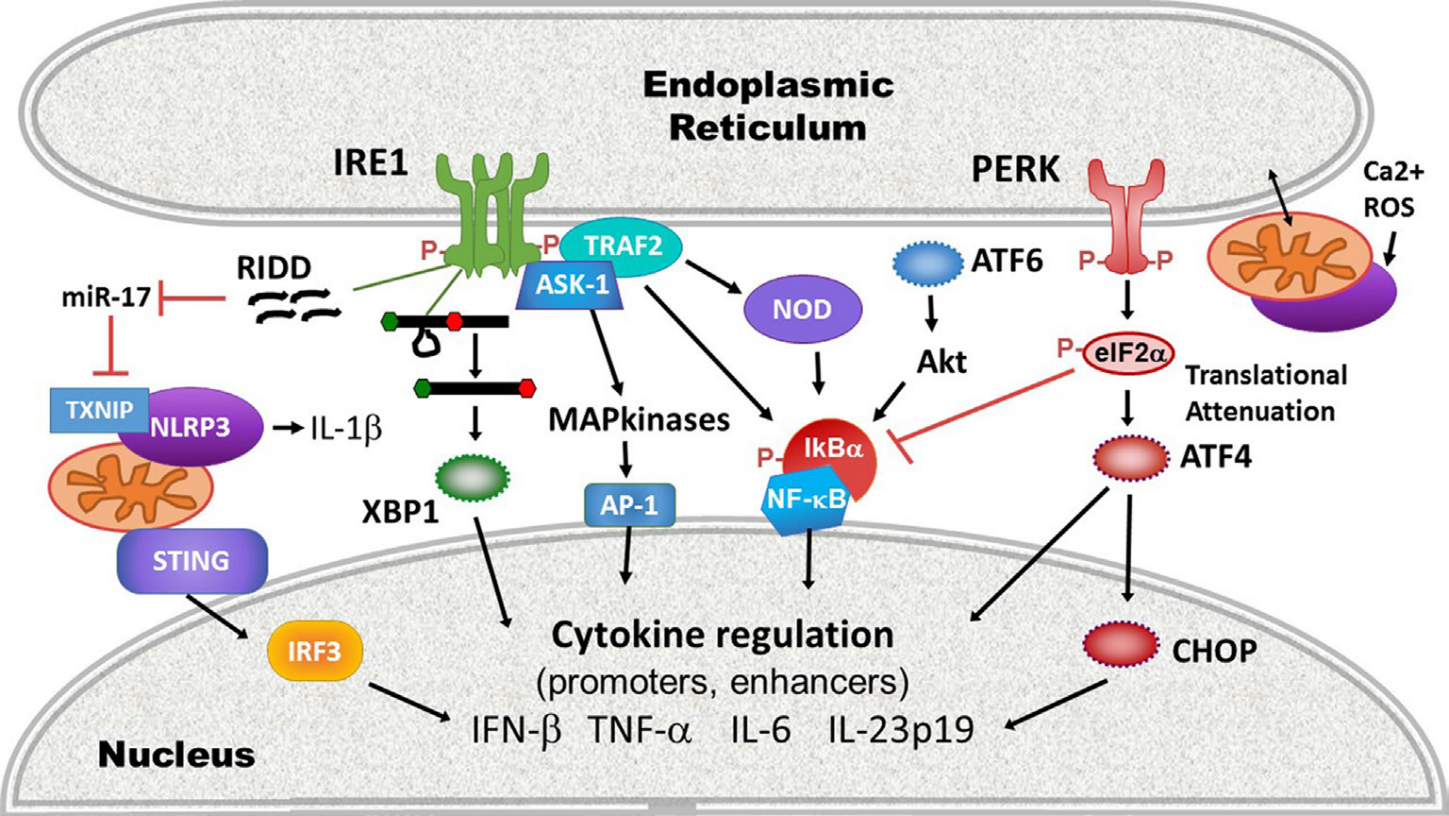
Intersections of endoplasmic reticulum (ER) stress/unfolded protein response (UPR) and immune signaling. ER stress and the UPR impact innate immune signaling and cytokine production on many levels between pathogen-sensing pattern recognition receptors (PRRs) and ultimate cytokine production: (1) PRR activation: ER stress and the UPR activate multiple PRRs (purple) including stimulator of interferon gene (STING), NLRP3, and other inflammasomes *via* thioredoxin-interacting protein (TXNIP) upregulation and reactive oxygen species (ROS), and the NOD1/2 receptors. Much of this interaction occurs at the mitochondrial-ER interface, where released calcium (Ca2+) and ROS feed into PRR activation. (2) The UPR enhances inflammatory signaling pathways leading to mitogen-activated protein kinase activation [IRE1 shown here, but protein kinase R-like endoplasmic reticulum kinase (PERK) and activating transcription factor 6 (ATF6) also impact ERK and p38 activation], and inhibitory factor κB (IκB) phosphorylation and degradation. (3) Transcription factors: the UPR activates canonical pro-inflammatory and IFN-regulatory transcription factors activator protein 1 (AP-1), nuclear factor kappa-light-chain-enhancer of activated B cells (NF-κB), and IRF3. Core UPR generated transcription factors such as XBP1 and C/EBP homologous protein (CHOP) also directly stimulate cytokine production by binding cytokine promoter and enhancer elements.

More recently, evidence has suggested that beyond directly regulating transcription factors or cytokine promoters, ER stress also impacts the activation of upstream PRRs. For example, ER stress activates the inflammasome, thus promoting IL-1β production and potentially programmed cell death. IRE1 activation, possibly *via* RIDD, inhibits a micro-RNA, miR-17, that down-regulates the production of thioredoxin-interacting protein (TXNIP) (110, 111). Thus, ER stress rapidly increases TXNIP expression (111, 112). PERK also increases *TXNIP* expression *via* the induction of transcription factors carbohydrate-responsive element-binding protein and ATF5 (112). TXNIP associates with and activates the NLRP3 inflammasome at the mitochondria. NLRP3 in turn, in a caspase-2 and BH3 domain interacting agonist (Bid)-dependent mechanism, causes mitochondrial damage, cytochrome C release, and production of oxygen radicals that further stimulates inflammasome production of IL-1β (113). The IRE1-RIDD function has also been implicated in the generation of small RNAs that trigger RIG-I-dependent NF-κB activation (114). UPR-dependent mitochondrial damage and mitochondrial DNA release may also play a role in the activation of another cytosolic sensor STING: mitochondrial DNA triggers the molecule cGAS, which in turn generates a cGAMP ligand that stimulates STING (115). As noted, certain types of ER stress mobilize STING translocation and STING-dependent IFN production (101). However, the link between *ER stress*-dependent mitochondrial damage and STING activation remains speculative. ER stress is well poised to initiate mitochondrial ROS-dependent events that activate and amplify innate immune signaling: protein folding is an oxidative process (116). The UPR and ROS trigger calcium release from the ER through activation of the inositol-1,4,5-triphosphate (IP3) receptor and ryanodine receptor ER channels. ER and mitochondria are spatially juxtaposed at the mitochondria-associated ER membranes, where ER IP3R channels are linked *via* chaperones to mitochondrial voltage-dependent anion channels (117). Increased cytosolic calcium thus triggers ROS release from mitochondria, which induces increased levels of ER stress, resulting in a relentless feed-forward loop (116). Finally, the UPR also interacts with the cytosolic peptidoglycan receptors NOD1 and NOD2 to induce production of the pro-inflammatory cytokine IL-6. Activation of this NOD1/2-dependent pathway by thapsigargin or infection with *Brucella abortus* was suppressed by the general UPR inhibitor tauroursodeoxycholic acid (TUDCA) and the IRE1 kinase inhibitor KIRA6 (118). The proposed mechanism involves IRE1 kinase activation and recruitment of NOD-interacting proteins TRAF2 and receptor-interacting serine/threonine-*protein* kinase 2 (119). For a summary highlighting mechanisms at the intersection of UPR and cytokine induction, see Figure 3.

Sterile ER stress results in relatively low levels of cytokine production, particularly compared with PRR stimulation (74, 120). In the case of IFN-β, this is perhaps surprising, as ER stress activates the critical enhanceosome components NF-κB, AP-1, and IRF3. One possible explanation is that another signal is required (e.g., PRR ligation) for full phosphorylation of IRF3 at multiple sites (101). Alternatively, although multiple UPR pathways activate NF-κB, it may still be at a low level compared with that induced by PRR ligation. Another possibility, extending to other cytokines, is that PRR ligation may be required to generate certain transcriptional co-factors or epigenetic modifiers.

In contrast to situations involving either sterile ER stress or isolated PRR stimulation, subsequent PRR ligation of cells undergoing ER stress has profound consequences for inflammation. Specifically, induction of ER stress has the capacity to render cells exquisitely sensitive to PRR stimulation, resulting in dramatically synergistic production of certain cytokines. This synergism has been demonstrated using pharmacologic UPR inducers, XBP1 overexpression, and misfolding proteins (74, 104, 120–122). Prominently increased cytokines include IL-6, TNF-α, IL-23, and IFN-β. In the cases of IL-6, TNF-α, and IFN-β, synergy appears to be XBP1 dependent but for IL-23, it is CHOP-dependent, consistent with their detected binding to specific cytokine gene regulatory elements (74, 102, 104). ER stress may also enable cells to produce IL-1β in response to TLR4 ligation in a TRIF (TIR domain containing adaptor protein inducing interferon beta)-dependent and caspase 8-dependent, but XBP1 and CHOP independent manner (123). Synergy is not the invariable outcome of PRR stimulation of stressed cells but may depend upon the context. In ischemic preconditioning, which induces ER stress, inflammatory cytokine production is blunted, possibly *via* ATF3 induction or decreased NF-κB activity (106, 124).

Direct ligation of PRRs on the other hand, in the absence of a specific ER stressor, appears to partially activate UPR signaling pathways and selectively suppress others. Woo et al. reported that TLR3 or TLR4 stimulation suppressed subsequent ER stress-induced ATF4 and CHOP activation (but not upstream PERK or eIF2α phosphorylation) in a TRIF-dependent manner (125). LPS suppression of CHOP limited apoptosis (126). Stimulation of TLR2 and TLR4 activates IRE1 sufficiently to induce XBP1 mRNA splicing and binding of XBP1 to cytokine promoters. Interestingly, in this setting the nominal XBP1 UPR targets genes (e.g., *ERdj4*) were not transcribed. TLR signaling did not trigger the other two UPR pathways, as assessed by PERK phosphorylation and ATF6 cleavage, and inhibited tunicamycin-dependent upregulation of CHOP and the ATF6 target BiP. Canonical TLR signaling pathways and ROS appear to be involved in TLR-induced XBP1 splicing, as NOX2, TRAF6, and TLR adaptors myeloid differentiation primary response 88 (MyD88) and/or TRIF were all required (74). As another example of partial UPR activation and modification, viral infections that release dsRNA stimulate PKR, eIF2α phosphorylation, and GADD34 induction, in a TRIF-dependent manner. Interestingly, in the setting of virus/dsRNA, GADD34 relieves the translational inhibition of IFN-β and IL-6, but not global translation (127, 128). The basis of this specificity, or the resistance of global translational reversal remains unclear. Likewise, it is not yet understood why TLR4 induced XBP1 would promote the production of cytokines, but not its nominal chaperone targets. This phenomenon of partial UPR signaling and modulation in response to PRR ligation has been termed the “microbial stress response” pathway (129). As a net result, PRR adaptation of the UPR machinery potentially boosts cytokine production while avoiding the apoptotic sequelae of a fully engaged UPR.

One other mode of UPR-cytokine cross-talk occurs between cells rather than within individual cells. Surface translocation of calreticulin in cancer cells due to ER stress enhances immunogenicity and phagocytic uptake by dendritic cells—an immunostimulatory “eat me” signal (130). In a subsequent study, thapsigargin but not tunicamycin treatment of fibroblasts increased surface calreticulin expression and phagocytic uptake by co-incubated dendritic cells, suggesting the type of ER stress may be important. Interestingly, incubation of these thapsigargin-treated fibroblasts with bone marrow cells augmented LPS-induced production of pro-inflammatory cytokines such as IL-1β, IL-12, IL-23, and TNF-α (75).

## Implications for Viral Infections

Intracellular infections provide a stage where ER stress interacts with signals from multiple PAMPs and DAMPs. The impact of the UPR on host-pathogen interaction has been increasingly recognized in viral, bacterial, and parasitic infections. UPR in the setting of parasitic infections has been reviewed recently and will not be discussed below (131).

The dramatic synergy observed by multiple groups between UPR and PRR signaling in the induction of type I IFN has particularly compelling implications for viral infection where the IFN response forms the capstone of host resistance. Viruses notoriously sabotage IFN production in a variety of ways. Several viruses interfere with the signaling leading to IRF3 activation or association with CBP/p300 (132–134). For instance, Dengue virus infection cleaves STING and also targets its upstream ligand-generator cGAS (135, 136). Other viruses target the type I IFN receptor IFNAR for proteolytic degradation (137). Paramyxovirus V proteins target STAT1 and STAT2 for proteolytic degradation (138). Therefore, one could speculate, that given all the viral obstacles to mounting an effective IFN response, even a partial UPR with XBP1 splicing or GADD34 induction to promote IFN-β transcription and translation might improve the odds.

Viruses induce ER stress through multiple mechanisms: during viral infection, cells dramatically increase protein production to manufacture new progeny virus. Some viruses reorganize the ER to develop replication platforms (e.g., Hepatitis C virus, coronavirus), and disrupt ER-Golgi trafficking (e.g., Picornavirus) (139–142). Viral infection also generate ROS. Beyond the host’s direct response to ER stress, the catalog of viral proteins that induce or manipulate UPR pathways has grown exponentially. One could envisage how the UPR could be both helpful and harmful to viral infection, even aside from any effects on the anti-viral IFN program. On the one hand, adaptive pathways within the UPR could enable host cells to survive the inordinate stress of significantly increased viral protein production by significantly increasing ER capacity. However, both translational inhibition and ERAD could diminish viral protein production. Premature UPR-related apoptosis could also limit viral replication and spread.

In recent reviews, 35 animal viruses and several plant viruses have been reported to provoke ER stress and/or UPR induction (143, 144). Viruses vary greatly in their capacity to both induce and inhibit individual UPR pathways. Multiple RNA viruses (e.g., Dengue virus, Hepatitis C, Coxsackie B3, and SARS coronavirus) and DNA viruses (Ebstein Barr virus, Hepatitis B) induce all three UPR signaling axes (65, 114, 143, 145–150). Several viruses have been reported to induce two UPR axes, for instance IRE1 and PERK (Sindbis) or IRE1 and ATF6 (Influenza A, Chikungunya), whereas some may induce only one arm [e.g., ATF6, lymphocytic choriomeningitis virus (LCMV)] (151–153). Different aspects of the UPR may also prevail at specific times during the viral life cycle (145). The basis for this selective activation is not well understood but may depend upon specific viral factors and intracellular lifestyle.

Viruses have co-evolved multiple mechanisms to manipulate specific UPR pathways to avoid some of the potentially detrimental effects of UPR induction. For instance, several viruses encode GADD34 homologs: the Herpes Simplex Virus 1 product γ_1_34.5 forms a complex with protein phosphatase 1, which dephosphorylates eIF2α, thus limiting translational inhibition (154). Further, γ_1_34.5 contributes to viral resistance to IFN-α/β (155). The African swine fever virus DP71L functions similarly, inhibiting induction of ATF4 and CHOP (156). Japanese encephalitis virus induces RIDD to enhance replication, but intriguingly appears resistant to the RNAse activity (157). Herpes Simplex Virus UL41 protein suppresses XBP1 mRNA induction and splicing, possibly to decrease ERAD (158). There are also examples of viruses (e.g., Hepatitis C) that are permissive for XBP1 splicing, but prevent induction of XBP1’s nominal UPR gene targets, which would include ERAD proteins such as ER degradation-enhancing α-mannosidase-like protein EDEM (159). This separation of XBP1 splicing and UPR target induction is reminiscent of the TLR-induced XBP1 disjunction. In these cases, it would be interesting to determine if the “blocked” XBP1s could still synergize in promoting IFN or pro-inflammatory cytokine production.

Modulation of the UPR appears to have varying effects, depending upon the virus and the type of UPR inhibition used. Viruses often induce pathways that enhance their replication. For instance, LCMV induces ATF6 activation, and cells defective in Site-2 protease produce lower titers of infectious virus (153). Likewise, blocking IRE1 with pharmacologic agents inhibits Influenza A replication (160). There are also multiple examples of viruses where the UPR appears to limit replication, suggesting a contribution to host defense. For instance, PERK is required for control of Dengue replication and pharmacologic eIF2α phosphorylators exhibit anti-viral activity (145, 161). Similarly, West Nile virus replicates at much greater titers in the absence of pro-apoptotic CHOP (162). Together, these studies support a general, but not universal concept that the IRE1 and ATF6 pathways are more likely to benefit virus, but the PERK pathway supports host defense. As an example where integrated stress response benefits virus, HIV induced ATF4 directly promotes HIV transcription through its long terminal repeat (163).

Although the UPR limits some viral infections, direct evidence for the role of the UPR in promoting type I IFN or other inflammatory cytokines during viral infection has been limited. It can also be difficult to tease apart cytokine vs. other effects of UPR modulation. For instance, in a Dengue model, induction of the UPR with a BiP inhibitor increased activation of IRF3 and NF-κB. However, it was not clear if these transcription factor effects contributed to anti-Dengue activity (164). There is some evidence viruses induce collateral damaging inflammation *via* UPR activation. For instance, the Hepatitis B protein HBx induced inflammatory cyclooxygenase 2 *via* an eIF2α-ATF4 pathway (114). Dengue virus-induced PERK/Nrf2 activation enhanced TNF-α production *via* increases in c-type lectin domain family 5, member A (CLELC5A), thus exacerbating pathology in a mouse model (165). Regarding IFN, in dendritic cells, XBP1 overexpression enhanced IFN-β production and markedly suppressed Vesicular stomatitis virus replication (122). In murine embryonic fibroblasts, GADD34 was required for dsRNA induced IFN-β and IL-6 production and resistance to Chikungunya virus. *In vivo*, IFN-dependence upon GADD34 appeared age-specific: adult mice were resistant to Chikungunya. However GADD34−/− neonates produced greatly diminished IFN-β in response to infection and rapidly succumbed (127). These two studies support a role for the UPR or microbial stress response pathways in supporting IFN and anti-viral immunity. However, viruses can also manipulate the UPR to limit IFN production. For instance, vesicular stomatitis virus and hepatitis C virus target IFNAR for proteolytic degradation *via* a PERK-dependent pathway, and this pathway appeared to enhance viral infection (137). Hepatitis C activation of UPR-autophagy pathways, including induction of CHOP and autophagy protein 5, also limited IFN-β production (166). Overall, the precise role of UPR pathways in supporting or limiting IFN or other cytokine production during viral infection, and the ultimate effect on pathogenesis remain important areas for further investigation.

## Implications for Bacterial Infections

The study of the UPR in bacterial infections is much younger and less well developed than for viral infection, but the complexity of bacterial lifestyles promises many interesting variations on the interactions between host UPR and immunity. The list of bacteria inducing UPR pathways through their intracellular lifecycles or elaboration of bacterial products is steadily growing. Regarding bacterial products, Subtilase toxin, produced by Shiga endotoxic *Escherichia coli*, cleaves BiP, thus initiating all three arms of the UPR (167, 168). Interestingly, this UPR activation may either promote apoptosis, or dampen NF-κB responses and endotoxic pathology at subcytotoxic doses (169, 170). Listeriolysin O, produced by *Listeria monocytogenes*, also induces all three axes of the UPR (171). The current mechanism is unknown, but may involve depletion of intracellular calcium stores (172). Cholera toxin selectively binds IRE1, activating its RIDD activity (173). *Brucella abortus* secretes a factor VceC *via* its type IV secretion system that binds BiP and selectively induced IRE1activation (174). Interestingly, when ectopically expressed, several other *Brucella* type IV secretion system substrates also appear to accumulate in the ER, inhibit protein secretion and induce varying amounts of ER stress (175).

Beyond secretion of ER/UPR modifying factors, several pathogens form intimate spatial relationships with the ER during their intracellular lifecycle. For instance, *Legionella* and *Brucella* traffic in the endosomal pathway, preventing full phagosome-lysosome fusion, and establish replicative vacuoles within ER-derived compartments (176, 177). *Chlamydia* containing inclusion compartments also contact the ER (178). Intriguingly, reports of the interactions of these three ER-localized pathogens with ER stress responses have varied. One group reported that persistent (non-productive) *Chlamydia* infection induced transient BiP upregulation and eIF2α phosphorylation but not ATF6 cleavage or XBP1 splicing (179). However, in another study, *Chlamydia* stimulated “robust” IRE1 activation and XBP1 splicing, and induced CHOP in a GCN2-dependent manner (180). *Legionella* actively inhibited XBP1 splicing *via* bacterial translation elongation inhibitors (181). *Brucella* infection induces pronounced activation of UPR pathways. Within 24–48 h of infection, *Brucella* causes massive restructuring of the ER marked by condensation, fragmentation, and vacuolization (176, 182). This restructuring is mediated, at least in part *via* a microtubule stabilizing factor produced by *Brucella*, TcpB, which also has UPR-inducing properties (182, 183). Although the UPR induced by *B. melitensis* involves all three axes, with prominent CHOP induction, the *B. abortus* triggered UPR appears more targeted in scope (174, 182). Interestingly, the UPR appears to benefit *B. melitensis* replication in that targeting IRE1 with RNAi in a *Drosophila* S2 cell line or in IRE1−/− fibroblasts, or treatment of macrophages with the general UPR inhibitor TUDCA all diminished replication (182, 184). The UPR may help the host cell to survive the tremendous structural insult to its protein producing factory through its adaptive pro-survival ER stress coping mechanisms. The UPR also induces autophagy through multiple pathways, thus providing increased nutrients to “feed” the bacteria (185). Autophagy may also promote cell–cell spread (186). In contrast to *B. melitensis, B. abortus* replication was not affected by TUDCA (118). The basis for this species difference in UPR induction and consequence is not clear.

Several lines of evidence support a role for the UPR in innate immune sensing of bacterial infection and control of infection or collateral inflammation. The cytokine response to *Chlamydia* involves multiple ER stress pathways: CHOP critically contributed to *Chlamydia*-induced IL-23 production (104). Chlamydia also induced PKR-dependent IFN-β through a mechanism requiring TLR4 and IRE1 RNase activity. Interestingly, this TLR4 activity may limit CHOP induction, stressing the importance of the multiple innate immune and ER stress inputs that impact cytokine production during infection (180). XBP1 deficiency significantly decreased TLR2-dependent TNF-α and IL-6 responses to *Francisella in vitro*. Furthermore, XBP1 conditional knockout mice infected with *F. tularensis* exhibited greater organ disease burden (74). UPR augmentation of cytokine production may be particularly important in *Brucella* infection because of the unusually low endotoxicity of its LPS, as well as the sabotage of TLR signaling by TIR-domain analog-containing bacterial factors (e.g., TcpB) (187, 188). In *B. abortus* infected macrophages, VceC and IRE1 was required for optimal IL-6 responses *in vitro* (174). In a subsequent study, this same group implicated the NOD1/NOD2 PRRs downstream of ER stress in *Brucella*-stimulated IL-6 production (118). *In vivo*, the VceC mutant stimulated much less splenic IL-6 production, despite similar bacterial burden. Furthermore, in an inflammatory abortion model, the VceC mutant, TUDCA treatment, or NOD1/2 deficiency all decreased placentitis, placenta IL-6 expression, and increased mouse pup survival (118). Thus, ultimately, the net benefit of UPR-supported inflammatory responses during infection may represent a balance between augmented host sensing of infection, containment, and collateral inflammatory damage.

## Implications for Autoimmunity and Autoinflammatory Diseases

The UPR potentially enhances host responses to invading pathogens by boosting PRR signals. However, the down side to immune augmentation is the capacity to cause pathologic cytokine production, even in the absence of infection. Aberrant cytokine production plays a critical role in fomenting inflammatory disease, as attested to by the tremendous clinical utility of cytokine blocking antibody therapies. Cytokine targeting therapy has been remarkably effective in both autoimmune disease [e.g., rheumatoid arthritis (RA)], where “self” autoantigens play key roles in disease pathogenesis, as well as autoinflammatory diseases, which are driven primarily by abnormalities in cytokine production [e.g., TNF-receptor-associated periodic fever syndrome (TRAPs) and cryopyrinopathies] (189). Some of the diseases discussed below [inflammatory bowel disease (IBD), spondyloarthritis (SpA)], although not a result of a monogenic cytokine dysregulation, also have prominent autoinflammatory features. For instance, in mouse models, exogenous expression of IL-23 (generated by genetic minicircle infusion) reproduces many of the clinical features of SpA, including sacroiliitis, enthesitis, and inflammatory skin disease (190). General overexpression of human TNF in mice phenocopies RA, whereas a stabilized TNF-α in mice (TNFΔARE) produces aggressive widespread (polyarticular) joint disease and Crohn’s like IBD, with arthritis occurring independently of T or B cells (191–193). In humans, genome wide association studies in polygenic autoimmune and autoinflammatory disorders have identified numerous associations with polymorphisms in cytokine or cytokine-regulatory genes (194–196). Thus, given the centrality of cytokine production in these inflammatory diseases, as indicated by clinical data, mouse models, and genetic studies, ER stress could theoretically have a major impact on disease pathogenesis. Indeed, the UPR has been implicated in an increasingly greater number of inflammatory diseases. A few themes will be highlighted below, including linkage of UPR components to polygenic autoimmune diseases, diseases of altered ER function, misfolding protein diseases, and autoimmunity in highly secretory cells.

Inflammatory bowel disease results from the aberrant, over-exuberant response to endogenous gut flora (197). Further, the association with *NOD2*, the first major gene linked to IBD, implicates innate immunity in the abnormal gut inflammation (198, 199). IBD is also one of the first polygenic disease to be genetically linked to UPR components (200). Specifically, a hypomorphic allele of *XBP1* increases risk of developing IBD. XBP1^ΔIEX^ mice, lacking XP1 in intestinal epithelial cells, develop spontaneous mild enteritis and are more susceptible to Dextran sodium sulfate-induced colitis (an experimental IBD model) (200). Autophagy or the process of “self-eating” interacts with the UPR on multiple levels, in that the UPR induces autophagic pathways and autophagy may limit the UPR (185). Interestingly, in the case of IBD, *ATG16L1*, encoding a core autophagy effector, also associates with IBD in human genetic screens, and ATG16L1^ΔIEX^ mice develop spontaneous Crohn’s like ileitis (201–203). ATG16L1^ΔIEC^ and XBP1^ΔIEC^ double knockout mice develop very severe colitis, suggesting a functional synergy between defective autophagy and UPR in predisposing to colitis (202). Part of the role of the UPR in colitis appears to be in support of gut-protective secretion: XBP1 supports Paneth cell development and function (200). However, there is also a more direct inflammatory consequence of XBP1 deletion. Through an unclear mechanism, XBP1 deficiency results in hyperactivation of IRE1. ATG16L1 deficiency in gut intraepithelial cells independently results in increased IRE1, related to defective IRE1 clearance by autophagy (203). Increased IRE1 kinase activity induces augmented NF-κB activation and thus pro-inflammatory cytokine production. Indeed, gut deficiency of IRE1 or TNFR1 relieves the XBP1^ΔIEC^ inflammatory phenotype (202). Mucin production maintains the barrier between gut flora and epithelial cells; “Winnie” and “Eyeore” mice expressing misfolding Mucin2 have a deficiency in mucin production, exhibit increased gut permeability and strong UPR induction, and develop gut inflammation (innate immune and Th17) (204, 205). Two other ER genes have also been linked to IBD in humans, anterior gradient 2 (*AGR2*), encoding a protein disulfide isomerase, and Orosomucoid-like 3 (*ORMDL3*), which regulates ER calcium and induces UPR pathways (206–209). Agr2−/− mice develop severe spontaneous ileocolitis associated with defective mucin folding and ER stress (210). At this time, it is not clear how ORMDL3 regulates gut inflammation. Together these studies suggest that the UPR-autophagy interaction regulates the extent of inflammatory responses to gut flora and that defects in this axis predispose to IBD.

More recently, protein mishandling/altered trafficking at the ER has been identified as a monogenic cause of an autoimmune syndrome. Patients with mutations in *COPA* develop inflammatory interstitial lung disease with pulmonary hemorrhages, arthritis, autoantibody production, and renal disease (211, 212). *COPA* encodes a component of the COP I complex responsible for Golgi-ER retrograde transit and the syndromic mutations in this gene appear to disturb protein cargo recognition. *COPA* mutant cells display signs of ER stress with increased *BiP, ATF4*, and *CHOP* expression, although the precise mechanism linking this defect in retrograde transit with ER stress are not yet clear. The ER stress correlates with increased expression of IL-1β, IL-6, and IL-23 in immortalized B cells from these subjects, previously noted ER stress augmented cytokines (74, 104, 123). Perhaps as a result of increases in these specific cytokines, patients also exhibit an expansion of T helper 17 CD4+ T cells, a cell type implicated in autoimmunity (213). Interestingly, a number of these patients also have evidence for a type I IFN-regulated gene signature in their peripheral blood (214).

TNFR1-associated periodic fever syndrome is an autosomal dominant monogenic autoinflammatory disease that manifests with episodes of prolonged high fever, rash, abdominal pain, peri-orbital edema, and myalgia (189). Defective surface shedding of TNF receptors (and thus prolonged TNF signaling) was initially postulated as a pathogenic mechanism; however, several studies have shown that TNFR1-associated mutants form oligomers and aggregates in the ER, resulting in ER retention (215). Interestingly, these mutations were also associated with defective autophagy, and increasing autophagy with geldanamycin decreased IL-β production (216). Patient peripheral blood mononuclear cells (PBMC) expressed elevated levels of phosphor PERK and spliced XBP1 mRNA, but not increases in other UPR-associated transcripts. Their monocytes had increased ROS as well (217). Transfection of cells with mutant TNFR1 did not induce BiP or CHOP expression, suggesting the UPR is not a direct contributor (215). However, cells from patients expressing mutant TNFR1 displayed increased mitochondrial ROS production, which promotes inflammatory cytokine production (218). Thus, ER stress may link misfolding TNFR1 to inflammation *via* ROS.

Spondyloarthritis encompasses a group of genetically and pathologically related inflammatory diseases which manifest with axial (spinal) arthritis, enthesitis, uveitis, gut inflammation, and psoriasis (219). SpA is highly linked to an MHC protein HLA-B27 that misfolds during biogenesis: in patients with the prototypic SpA, ankylosing spondylitis, 80–90% of subjects are HLA-B27 positive vs 6% of the United States population (220, 221). Although ankylosing spondylitis is a polygenic disease, the presence of HLA-B27 accounts for the preponderance (67%) of the currently identified heritability, conferring an odds ratio of >50 (222, 223). This misfolding propensity and prolonged association with BiP in the ER results from specific amino acids in its peptide-binding B pocket and unpaired cysteines (224–226). The subtypes of B27 with differential disease association also exhibit variance in biochemical features including thermos-stability, folding rates, and intracellular aggregation (227–229).

Transgenic HLA-B27 expression alone is sufficient to drive an inflammatory disease analogous to SpA in susceptible rat strains, although disease requires very high-transgene numbers (230, 231). Interestingly, disease does not occur in germ-free rats, but requires microbiota (232). Although there are many reasons why this might be the case, in light of the current discussion, one could speculate that microbiota may also be required to provide PRR signals that synergize with ER stress. Interestingly, CD8+ T cells are dispensable for disease development in rats, suggesting another property of HLA-B27 besides its antigen-presenting capacity may be important in driving disease (233). Bone marrow-derived macrophages from HLA-B27, but not HLA-B7 transgenic rats showed evidence for a UPR gene signature, particularly when class I MHC was acutely upregulated by cytokines such as TNF-α and/or IFN (234). These ER stressed macrophages responded to TLR agonists with greatly increased type I IFN *in vitro* (120). Interestingly, the bone marrow macrophages from the diseased B27 transgenic animals displaying a UPR gene signature also exhibited a very prominent IFN signature (234). However, the role of IFN, if any, in SpA has not been established. The inflamed colons in diseased animals exhibited upregulation of UPR target genes, along with increased IL-23, IL-17, IFN-γ expression, and expansion of Th17 cells (121). In an effort to more directly address the role of the UPR in these rats, one study interbred HLA-B27 transgenic rats with human beta-2 microglobulin overexpressing rats to stabilize and aid in HLA-B27 folding. This breeding did indeed reduce misfolding in Con-A stimulated splenocytes, although macrophages and tissue UPR were not assessed (235, 236). Surprisingly, these animals developed more severe arthritis, without changes to their colitis. This study suggests the role of HLA-B27-linked UPR may be discordant in the joints and the gut during SpA and raises further questions regarding HLA-B27 misfolding, UPR, and disease pathogenesis.

Although HLA-B27 can induce a UPR, it is not clear this property is the culprit in human subjects expressing at most two copies of the MHC molecule. HLA-B27 also forms surface dimers that can stimulate IL-17 producing cells, providing an alternative mechanism (237). Studies examining UPR in human subjects have yielded inconsistent results: increased BiP has been observed in knee fluid macrophages from ankylosing spondylitis patients (238). PBMC monocytes have been reported to express higher levels of UPR target genes, although other groups have reported a lack of UPR in PBMC and synovium (239, 240). Blood-derived macrophages from ankylosing spondylitis patients produce increased IL-23 in response to LPS without increased UPR target gene expression (241). Misfolded HLA-B27 has been detected in gut biopsies from SpA patients, but associated with activation of autophagy rather than UPR (242). Also, not all SpA (or even ankylosing spondylitis) patients are HLA-B27 positive. Interestingly, in a mouse model with altered autoreactive T cell repertoire, curdlan or zymosan treatment induces an SpA-like disease with enteritis, sacroiliitis, enthesitis, and psoriatic skin inflammation (243, 244). This disease model is also cytokine (IL-23 in particular) and gut microbiome-dependent (245). Interestingly, the inflamed colons from these animals showed evidence of UPR target gene induction (243). Thus, misfolding HLA is not an absolute prerequisite for UPR induction in SpA pathogenesis. These observations also raise the possibility that the UPR may be an integral part of the developing inflammatory process and not just the inciting event.

Myositis is another rheumatologic entity linking aberrant MHC, a type I IFN signature and the UPR. This group of diseases includes dermatomyositis, inclusion body myositis and dermatomyositis. Muscle biopsies from these patients exhibit either CD4+ or CD8+ T cell infiltrate, along with macrophages, and dendritic cells, implicating adaptive and innate immunity (246). Both peripheral blood (dermatomyostis and polymyositis) and muscle biopsies (dermatomyositis) showed evidence for a type I IFN signature and the blood signature correlated with disease activity (247–249). Muscle biopsies from autoimmune myositis patients and inclusion body myositis patients also showed evidence for UPR activation, supported by increased expression of BiP, PERK, GADD 153, ATF3, and chaperones such as grp94, calnexin, calreticulin, and ERp72 (250, 251). Myocytes do not typically express abundant MHC class I, but class I molecules are highly expressed in muscle from these patients, in conjunction with elevated ER stress markers and NF-κB activation (250, 252). Although the link between aberrant MHC expression and ER stress driven inflammation in human cells is mainly correlative, in mice, transgenic overexpression of H-2Kb in skeletal muscle drives an inflammatory myositis phenotype associated with autoantibodies and ER stress (253, 254). Myositis was particularly severe in young mice compared with adults (254).

Besides myositis, an increasing number of rheumatologic conditions appear to be associated with a type I IFN gene signature. This list prominently includes systemic lupus erythematosus (SLE), Sjogren’s disease and systemic sclerosis (255). Moreover, in SLE, the gene signature also correlates with disease activity (256). Outside of plasma B cell development, current evidence for UPR activation in SLE is meager: lupus PBMC showed increased XBP1s but decreased expression of IRE1, PERK, and CHOP (257). T lymphocytes from SLE patients may be more susceptible to ER stress-induced apoptosis, related to defective BiP and autophagy (258). On the other hand, anti-double-stranded DNA antibodies, which are characteristic of lupus, induced both ER stress and cytokine production from human kidney mesangial cells (259). In systemic sclerosis, PBMC from patients showed upregulation of BiP, ATF4, ATF6, XBP1s, along with increased DNAJB1 and IFN-related genes. Furthermore, ER stress markers correlated with disease severity (the presence of pulmonary arterial hypertension) and IL-6 levels (260). Systemic sclerosis involves overproduction of pro-fibrotic cytokines, such as TGF-β, aberrant tissue deposition of collagen, and differentiation of fibroblasts and epithelial cells into myofibroblasts (261). TGF-β increased ER stress in lung fibroblasts, as evident by BiP, ATF6, and XBP1s induction, and also increased expression of α-smooth muscle actin and collagen. Indeed, ER stress may mediate the induction of the myofibroblast proteins, as the chemical chaperone 4-PBA inhibited TGF-β induced α-smooth muscle actin and collagen induction (262). The IRE1α endonuclease pathway also regulated TGF-β driven myofibroblast differentiation in human cells (263).

Finally, autoimmunity frequently targets physiologically highly secretory cells. Autoimmune thyroid diseases are the most prevalent autoimmune conditions and thyrocytes pump out abundant thyroglobulin (264). Melanocytes mount a UPR to cope with melanin production and become targets in vitiligo (265). In the pancreas, β-cells are insulin-producing factories that increase production up to 25-fold in response to glucose (266). In vitiligo and diabetes, CD8+ T cells appear to kill their cellular targets very specifically, without damage to neighboring tissue (265). However, although the autoimmune destruction is carried out by adaptive immune cells, pro-inflammatory cytokine production plays a critical inciting role. The T cell recruiting IFN-regulated chemokine CXCL10 is critical for the development and maintenance of vitiligo (267). In diabetes, IL-1β and IFN-γ induce β-cell apoptosis by stimulating reactive oxygen and nitrogen species (268). Beta-cell death generates autoantigen. Beta-cells also secrete chemokines CXCL10 and CXCL9 that recruit T lymphocytes to the islets (269).

**Figure 4:**
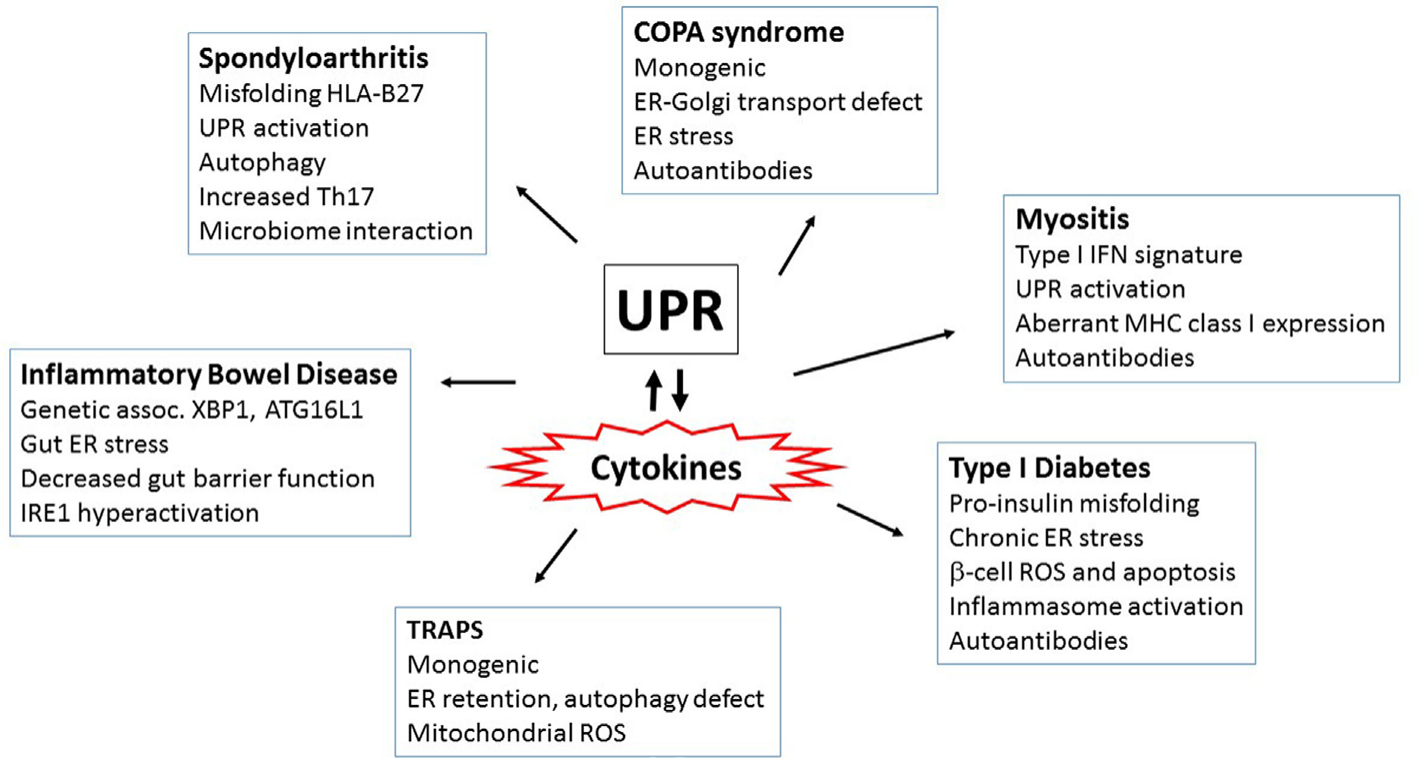
Autoimmune and autoinflammatory diseases involving the unfolded protein response (UPR) and endoplasmic reticulum (ER) stress. Aberrant or excess cytokine production plays a key role in driving autoimmune and autoinflammatory disorders. Interestingly, ER stress and/or the UPR has been increasingly implicated in these same diseases. Thus, the multiple mechanisms by which the UPR interacts with cytokines (both cytokines inducing ER stress and UPR regulating cytokine production) have repercussions for the pathogenesis of inflammatory diseases. Several of the diseases highlighted in this review and the prominent features surrounding ER stress and cytokine induction are in boxes. More autoinflammatory disorders are to the left and autoimmune on the right.

Endoplasmic reticulum stress and the UPR interweave through diabetes pathogenesis on multiple levels. The UPR is absolutely required for basal pancreatic function; PERK−/− mice die early from diabetes and exocrine pancreas failure (28). IRE1/XBP1s activity was also required for glucose-stimulated increases in insulin production and protection from oxidative stress (270). Islets from both diabetes-prone non-obese diabetic (NOD) mice and early human diabetes patients exhibited signs of chronic ER stress with increased CHOP expression and decreased pro-adaptive XBP1 and ATF6 (271, 272). Furthermore, treatment of the NOD mice with TUDCA restored UPR function and markedly protected NOD mice from the development of diabetes (271). Pro-inflammatory cytokines, particularly TNF-α, IFN-γ, and IL-1β induced ER stress (particularly CHOP upregulation) in β-cells (273, 274). TUDCA also protected islet cells from cytokine-induced JNK activation and apoptosis (274). The pro-insulin molecule is prone to misfolding, and human mutations that increase misfolding cause infantile diabetes (275, 276). In the Akita mouse model of diabetes, a mutation in the *Ins2* gene that prevents proper proinsulin folding (C96Y) leads to early onset diabetes associated with ER stress. CHOP deficiency delayed diabetes onset in this model by 8–10 weeks (277). In β-cells, activation of IRE1 promoted increased TXNIP expression *via* miR-17 degradation. TXNIP induction also depended upon PERK. ER stress-induced IL-1β and TXNIP-dependent apoptosis in islets. In THP-1 monocytes, induction of IL-1β depended upon TXNIP and NLRP3 (111). Other studies have also linked NLRP3 and islet IL-1β in type 2 diabetes (278). In the Akita model, deletion of TXNIP protected against β-cell apoptosis and ameliorates diabetes severity (111). Interestingly, NLRP3 deficiency did not prevent diabetes in Akita mice, suggesting other inflammasomes or TXNIP activities may play a role (279). IRE1α has also been linked to the development of diabetes in the NOD mice: targeting the ABL kinases that hyperactivate IRE1 (and thus decreasing IRE1 activity) reversed diabetes in NOD mice (280). These studies provide tantalizing clues that link diabetes and IRE1 activation; however, the connection between ER stress and early cytokine production and apoptosis in these autoimmune conditions remains an open area of investigation. For a summary of the autoimmune and autoinflammatory disorders highlighted above, see Figure 4.

## Conclusion and Perspectives

In summary, the ER plays an indispensable role in cell function and is sensitive to many types of stress; the ER is thus perhaps uniquely poised to transmute significant threats to cell function into amplified immune responses. Because of this role in sensing threats that perturb proteostasis, ER stress has been referred to as a “dyshomeostatic DAMP” (14). From an evolutionary perspective, it may not be mere serendipity that UPR molecules exhibit homology with ancient cytosolic anti-viral proteins, PERK with PKR and IRE1 with RNaseL (281, 282). Numerous pathways interweave the UPR and inflammation, making the ER an effective nidus for promoting sterile inflammation or dramatically amplifying PRR responses. Specifically, the UPR regulates cytokine production through a variety of mechanisms extending from PRR sensing to inflammatory signaling and cytokine transcription factor activation. During infection, the UPR may enable cells to titer the degree of threat, providing greater cytokine responses for threats that impact cell function vs. those that merely stimulate PRRs. The UPR may also enable infected cells to sense invasion by pathogens that otherwise sabotage PRR signaling. Perhaps one of the costs of this inflammatory amplification is the potential for inappropriate activation in the absence of pathogens. The UPR has been increasingly implicated in the pathogenesis of a number of autoimmune and autoinflammatory conditions where cytokines play a central role. However, at this point, much of this is guilt by associations. Although the pieces are there (evidence for UPR, aberrant cytokine production), the exact causative relationships await further definition.

The material presented above raise a number of questions, ranging from mechanistic to teleological. Several questions surround the regulation of the different modes of IRE1 (kinase, RIDD, and XBP1 splicing) activation. Is degree of oligomerization critical or association with co-factors? Does XBP1 directly or indirectly limit kinase activity? Is this occurring *via* ERAD of IRE1? During TLR4 ligation how does XBP1 promote cytokine production but not its other UPR gene targets? Is this also related to co-factor or heterotypic binding? During viral infections, how does GADD34 promote IFN production and not translation of other targets? For that matter, how does Japanese encephalitis virus trigger RIDD but specifically avoid degradation? The relationship between the proposed microbial stress response and UPR also requires further clarification. Drawbacks to the TLR-mediated suppression of ATF6 and PERK include inhibiting cytokine promotion by these pathways (e.g., NF-κB activation) or adaptive pathways that enable cells to survive stress or commit apoptosis when infected. Infections may induce both ER stress and stimulate multiple PRRs. Perhaps the relative balance of PRR stimulation vs degree of ER stress sways the cell toward either UPR or microbial stress response.

Although the UPR *can* regulate cytokines, how much of a role *does* the UPR actually play in cytokine induction during infection and autoimmunity? Moving from the relatively clean results obtained with selective pharmacologic UPR agonists or PRR agonists to the “messy” reality of an intracellular infection or autoimmune disease has been challenging, related to the tremendous increase in complexity. Beyond cytokine regulation, the UPR heavily influences autophagy, nutrient mobilization, and cell death. These other effects of the UPR make it difficult to assign particular responsibility to its effects on cytokines. For instance, it is challenging to tease apart the direct effect of the UPR on viral replication vs. augmented IFN production. In autoimmunity, the UPR may critically regulate autoantigen generation (and presentation) or the basal function of immune type cells. This may be a deus ex machina concept, but perhaps the sheer number of intersections between cytokine regulation and the UPR and the magnitude of effect (e.g., log-fold for IFN) provide support for their significance in disease pathogenesis.

The availability of small molecule inhibitors or agonists for different UPR pathways has grown exponentially, driven by the interest in developing novel therapeutic approaches to cancer and autoimmunity. As an example of repurposed cancer drugs, proteosome inhibitors, which affect proteostasis (and thus ER function) and cytokine production, have demonstrated efficacy in murine lupus models (283, 284). UPR modulating agents may also be useful for intractable infectious diseases or vaccine development. Some of these UPR drugs have already moved to clinical trials. For instance, Celgosivir, which inhibits N-linked glycosylation, is undergoing a phase II trial for Dengue (285). Better understanding of the role of the UPR in specific settings will be critical for the judicious trial of these new therapies; given the complexity of UPR-immune interactions, carefully conceived pre-clinical studies may be necessary to gage the net effect of individual UPR modulating agents on specific infectious or inflammatory conditions. It will be important not to generalize, as the role of the UPR is likely to be highly context specific, even between species of pathogen. An example described above, *B. melitensis* and *B. abortus* have been reported to induce different degrees of UPR activation and blockade with TUDCA appears to have different effects on replication (118, 182). Also, it will be important to balance the anti-pathogenic effects of UPR modulation against the potential of disturbing physiologic UPR responses. Given the exciting clinical potential for UPR modulation, clarification of these issues has become a compelling mandate.

## Author Contributions

The author confirms being the sole contributor of this work and approved it for publication.

## Conflict of Interest Statement

The author declares that the research was conducted in the absence of any commercial or financial relationships that could be construed as a potential conflict of interest.
